# Editorial for the Special Issue on Micro/Nanophotonic Devices in Europe

**DOI:** 10.3390/mi14040861

**Published:** 2023-04-16

**Authors:** Luigi Sirleto, Giancarlo C. Righini

**Affiliations:** 1 Institute of Applied Sciences and Intelligent Systems (ISASI), CNR, 80131 Napoli, Italy; 2 “Nello Carrara” Institute of Applied Physics (IFAC), CNR, Sesto Fiorentino, 50019 Florence, Italy

Photonics has often been defined as the key technology of the 21st century. In recent decades, the overall trend of research and development (R&D) towards the miniaturization of devices and systems, pushed by the tremendous development of microelectronics, has led to continuous development of micro/nanophotonic devices. They have huge potential for low-cost, scalable production, as well as the integration of high-density components. Their evolution would enable advanced applications to emerge not only in optical processing and computing, but also in metrology, single-molecule sensing, imaging, microscopy, mid-infrared photonics, terahertz generation, microwave photonics, and biomedicine.

Many research centers worldwide are working on projects related to advances in micro/nanophotonic devices. Although it is challenging to define the borders of such a research area, it is certainly true that a significant number of scientific projects have been or are being carried out in Europe, often in collaboration with colleagues from abroad. The growth of such projects has certainly been facilitated by the multi-year programs funded by the European Commission; among the successful examples of cooperation projects at the European level, one can mention the regional photonics clusters, corresponding to national technology platforms, and the European Technology Platform Photonics21.

By examining the scientific production in the areas of microphotonics and nanophotonics, one can see (perhaps with some surprise) that the publications listed under the topic “microphotonics” are 20 times fewer (≈550) than those appearing under the topic “nanophotonics” (≈11,050), according to the Clarivate Web of Science™. If we refer, for the sake of simplicity, to the sole area of nanophotonics, and we filter the results on the basis of the authors’ country of residence (as indicated in each publication), the result is that 3778 publications, i.e., over 34% of the total, are authored by at least one scientist from one of the 28 countries of the European Community, plus Switzerland and the UK. The image in Figure 1 gives a graphical representation of the larger geographical contributions; it also makes the importance of the collaboration with abroad countries, such as USA and China, evident.

The present Special Issue (SI) of *Micromachines*, entitled “Micro/Nanophotonic Devices in Europe”, represents a weak effort to shed some light on the ongoing researches and the achieved results in the European laboratories. Although it is far from an exhaustive overview, this Special Issue presents a few examples of the potentiality of micro/nanophotonics devices in a relatively wide spectrum of applications.

In the first group of papers, three reviews are proposed, aiming to highlight the state of the art of integrated photonics and its applications in optical communications and optical processing.

Silicon photonics is currently a commercially established and yet fast-growing technology for communication systems. To monitor the status of fabrication processes and characterize the performance of photonic circuits after production, wafer-scale testing is an essential technology in a semiconductor production line. Various techniques can be used to couple the optical signal from optical fibers to photonic chips, such as prism coupling, butt coupling, end-fire coupling, and grating coupling. However, these techniques allow for the performance of the entire circuit to be measured during wafer-scale testing, although it is not possible to test any subsystems or individual devices on the photonic chips. Therefore, in order to routinely test some key sensitive subsystems and individual devices on the photonic chips at the wafer scale, erasable grating couplers and directional couplers are highly desirable. Germanium (Ge) ion implantation into silicon waveguides induces lattice defects in the silicon, which can eventually change the crystal silicon into amorphous silicon and increase the refractive index from 3.48 to 3.96. A subsequent annealing process, using either an external laser or integrated thermal heaters, can partially or completely remove these lattice defects and gradually change the amorphous silicon back into its crystalline form, therefore reducing the material’s refractive index. Keeping in mind this change in optical properties, various erasable photonic devices which can be used to implement a flexible and commercially viable wafer-scale testing method for a silicon photonics fabrication line were reviewed in the paper by Yu and others [1]. In addition, Ge ion implantation and annealing were also demonstrated to enable post-fabrication trimming of ring resonators and Mach–Zehnder interferometers (MZIs). Finally, a proof of principle of nonvolatile programmable photonic circuits with the Ge implantation technology was also implemented. It is certain that, based on the refractive index change associated with the aforementioned Ge ion implantation and annealing, the flow of an optical signal within the photonic circuit can be switched or rerouted using erasable directional couplers or trimmable MZIs [1]. 

The combination of integrated optics technologies with nonlinear photonics, which has led to the growth of nonlinear integrated photonics, has also opened the way for groundbreaking new devices and applications. In this Special Issue, two companion review papers, dedicated to nonlinear photonics and aimed at offering a broad overview, are proposed [2,3]. In the former, the main physical processes involved in nonlinear photonics applications are introduced, starting from traditional second-order and third-order phenomena and progressing to ultrafast ones (self- and cross-phase modulation, supercontinuum generation, and optical solitons). The applications, on the other hand, were made possible by the availability of suitable materials with high nonlinear coefficients, and/or by the design of guided-wave structures, which can enhance the materials’ nonlinear properties. A summary of the most common nonlinear materials was also presented, together with a discussion of the innovative ones. Silicon and related materials, such as SiN, a-Si, and SiC; glasses, such as silica, high-index glass, and chalcogenide ones; III–V semiconductors; lithium niobate; and, more recently, investigated materials such as tantalum pentoxide and vanadium dioxide were discussed, and their pros and cons were pointed out. Lastly, two-dimensional layered materials and zero-index media were also introduced [2]. The latter review aimed at describing the development of integrated photonics, which began with centimeter-long circuits, progressed through microphotonics, and finally arrived at nanophotonics. The first step, from the centimeter to micrometer scale, was motivated by the wish to investigate light behavior and its interaction with micro-objects on the microscale. The key challenges were reducing the sizes of optical devices and improving their performances. The main goal was to develop a reliable platform for dense integration. The second step, from micro to nano, was motivated by the demand that, in the near future, it is expected for devices to control light in very thin nanoscale layers or in nanoparticles of nonlinear material. Various waveguide structures, fabrication processes, and integration platforms were discussed. Finally, a brief, and certainly not exhaustive, overview of nonlinear photonics devices was given; for simplicity, the examples were classified into three groups, each characterized by a common goal, even if quite broad: all-optical digital devices, devices for all-optical processing, and nonlinear light sources [3]. 

Nanophotonics is an attractive field of recent development. In the second group of papers, two contributions were proposed: the first related to metamaterials and the second related to dielectric nanostructures. These are among the most promising approaches for nanophotonics devices.

Toroidal multipoles are a class of fundamental electromagnetic excitations that complement the more familiar electric and magnetic multipole families. Recently, metamaterials have provided fertile ground for the observation of toroidal dipole and higher-order multipoles through the ability to judiciously shape the meta-atom/meta-molecule geometry in the unit cell. In the referenced paper [4], a conductive meta-atom of toroidal topology was studied both theoretically and experimentally, demonstrating a sharp and highly controllable resonant response. Simulations were performed both for a free-space periodic metasurface and a pair of meta-atoms inserted within a rectangular metallic waveguide. A quasi-dark state (almost non-radiating) with controllable radiative coupling was supported, allowing for the linewidth (quality factor) and lineshape of the supported resonance to be tuned via the appropriate geometric parameters. The structure was fabricated with a 3D printer and coated with silver paste. Importantly, the structure was planar, consisted of a single metallization layer, and did not require a substrate when neighboring meta-atoms were touching, resulting in a practical, thin, and potentially low-loss system. Measurements were performed in the 5 GHz regime with a vector network analyzer, and a good agreement with the simulations was demonstrated.

Plasmon-based devices operating at an optical and near-infrared frequency have been demonstrated to reach extraordinary field confinement capabilities, with localized mode volumes of only down to a few nanometers. Although such levels of energy localization are substantially unattainable with dielectrics, it is possible to operate subwavelength field confinement by employing high-refractive index materials with proper patterning, e.g., photonic crystals and metasurfaces. In the last 20 years, optical surface modes, i.e., optical modes strongly confined at the truncation interface of planar dielectric multilayers (called Bloch surface waves (BSWs)), on flat and patterned dielectric multilayers have been investigated in many frameworks. In another paper [5], BSWs, as a viable alternative to plasmonic platforms allowing for easy wavelength and polarization manipulation and reduced absorption losses, are investigated. A computational study on the transverse localization of BSWs by means of quasi-flat Fabry–Perot microcavities, which have the advantage of being fully exposed toward the outer environment, was proposed. These structures are constituted by defected periodic corrugations of a dielectric multilayer top surface. The dispersion and spatial distribution of the cavity mode of BSWs were studied. In addition, the hybridization of BSWs with an A exciton in a 2D flake of tungsten disulfide (WS2) was also addressed. WS2 monolayers display a narrow and intense excitonic resonance at 2.03 eV that is well suited to promoting mode hybridization with BSWs. A strong coupling, involving not only propagating BSWs, but also localized BSWs, namely, band-edge and cavity modes, was pointed out.

The recent developments in biophotonics have pushed the application of photonic devices towards microscopy and imaging. The third group of papers [6,7,8] can be placed in this frame.

Long-focusing range beams (LFRB), also known as quasi-Bessel beams, are appealing due to their non-diffractive properties. Their use in various important applications, such as in optical manipulation, high-resolution lithography, and microscopy, can be enhanced if the focal spot has a submicron full width at half maximum (FWHM) diameter. Another desirable feature when working with LFRBs is the possibility to tune the light distribution without moving parts in the experimental set-up. A possible solution can be obtained through forming LFRBs by exploiting bilayers composed of weakly absorbing film and thicker layers possessing thermo-optical properties. The working principle is based on the phase change of an input Gaussian beam induced in the latter film via the thermo-optical effect, while the heating power is produced by the optical absorption of the former film. The phase-modified beam is transformed by an objective into a long focus with a submicron diameter. A difficult challenge is the fabrication of ultrathin thin absorbers with (1) high flat spectral transmission, (2) sufficient absorption in the Vis–NIR spectral region, (3) high and uniform heat conduction, and (4) high chemical and structural stability. In this context, particular attention is focused on the emerging 2D materials, thanks to their intrinsic characteristic of low effective thickness and interesting optical properties for photonics applications. The theoretical and experimental characterizations of an elastomeric polydimethylsiloxane/single-layer graphene (PDMS/SLG) axicon-like tunable device, which is able to generate diffraction-resistant submicrometric spots, were reported in [6]. The results demonstrated that in spite of its low absorption, the SLG can significantly raise its temperature and even deliver moderate optical pump powers by heating the PDMS sufficiently to push its temperature close to the tolerance limit (~470 K) and generating a remarkable refractive index gradient. The proposed thermo-optical device can, thus, be useful for the simple and cheap improvement of the spatial resolution on long focus lines.

Traditionally, in carrying out macro tensile tests, the strain components are measured by means of a strain gauge attached to the sample. Of course, for miniaturized specimens, these instrumentation devices may not be suitable due to the reduced sample size. A non-contact optical technique can be more suitable and more conveniently used for accessing full-field strains over the target region. For this purpose, several white-light and interferometric optical methods have been proposed in the literature. Among them, the Digital Image Correlation (DIC) has the merit to allow one to measure full-field kinematic quantities by tracking unique features of images taken at different deformation stages. The DIC technique uses a camera-lens optical setup, and it allows for variable sensitivity and resolution in strain measurements. Reference [7] described how the digital image correlation (DIC) technique may be coupled with mechanical tests to quantify the deformation of a material across the central region of interest for both macro and miniaturized specimens. The development of equipment dedicated to performing uniaxial, tensile–compression tests on miniaturized metallic specimens was presented. The experimental equipment, called MSTD (miniaturized specimen tester device), permitted the testing of specimens on a miniaturized scale, thus opening up a broad field of applications, one of them being compressive loading tests, as the equipment has the advantage of overcoming buckling effects. In addition to uniaxial tensile, compression, and cyclic tests, it is also possible and expected that equipment will adapt to other fundamental tests for metallic sheets, such as shear and ductile damage tests.

Non-contact current surface texture analysis techniques include coherence scanning interferometry, confocal microscopy, and scanning laser microscopy. A drawback of the currently employed surface texture analysis techniques is their inability to capture the full surface of a sample, thus leaving users restricted to being able to analyze only a limited area. A unique solution to the aforementioned drawback may be found in electrically tunable lenses (ETL), which possess the ability to alter their optical power in response to an electric signal. This feature allows such systems not only to image the areas of interest, but also to obtain spatial depth perception. The mechanism of ETL is achieved via two main approaches: local variations in the refractive index, which include liquid crystals, and controlling the shape of the lens with two main techniques that use electrowetting and shape-changing polymers. A low-cost, non-destructive, ETL-based imaging system for the 3D surface characterization of materials was described in reference [8]. The system was based on a polymer-based liquid ETL. The tuning of the voltage and feeding of the ETL enables the adjustment of the ETL focal power. Images were captured via a camera attached to the ETL system. The system as then employed to measure the amplitude, spatial, hybrid, and volume surface texture parameters of a model material (pharmaceutical dosage form), and these were validated against the parameters obtained using optical profilometry.

One of the most important and significant area of applications for photonics devices is sensors and actuators. The fourth group of papers [9,10,11] is related to this field of applications.

Optical fiber sensors have provided effective sensing devices for decades. A wide variety of configurations have been successfully reported, according to the development of optical fiber fabrication techniques. Single-mode–multimode–single-mode (SMS) fiber structures have received significant attention in the last years due to their unique spectral features, high sensitivity, easy fabrication, and potential low cost. The general sensing principle of an SMS fiber sensor is based on the self-imaging effect in a multimode waveguide, i.e., multimode interference. In this framework, a new and attractive approach is to consider specialty fibers. An interesting example is given by square-core fibers, which produce an optical beam with uniform intensity over the core area, because the shape of the core promotes mode mixing as light propagates through the fiber. In one of the papers included in this Special Issue [9], the sensing performance of a simple multimode-interference-based fiber sensor, containing a square-core fiber 30 cm in length, was investigated for temperature and strain measurement. In addition, a comparison to other specialty fibers, which were mostly circular core shapes, was also presented. The experimental results demonstrated that this fiber sensor was able to exhibit great potential for applications in strain-insensitive temperature measurement.

The conversion of light into mechanical work has been a hot topic for many years. This approach is of great interest because actuators can be temporally, spatially, and remotely controlled by light, which can be beneficial in various applications. One of the most investigated photo-responsive liquid crystalline molecules is Azobenzene, which can be used as a molecular engine in photo-mobile materials. The use of azobenzene molecules embedded as moieties in liquid crystal elastomers yields photo-mobile materials that efficiently convert light into mechanical output. In reference [10], the thermo/mechanical phenomena, regulating the actuation of three LC mixtures consisting of 100% azobenzene moieties, were investigated. The nematic temperature of the LC elastomers was measured and the PMPs were carefully characterized for their bending and speed capabilities. The results demonstrated that the nematic temperature of the LC mixture was greatly influenced by the ratio of the linear and cross-linker monomer; when their ratio was in favor of the linear monomer, the PMPs bent more and the process was faster compared to the other mixtures. Finally, it was shown that the thermal effects did not play a predominant role in the movement of the film, and that such material could be useful for designing polarization-selective switches.

Quartz-enhanced photoacoustic spectroscopy (QEPAS) is a well-known technique used for the detection of specific trace gases in complex mixtures. The high performances in terms of selectivity and sensitivity allow for the exploitation of this technique in a wide range of applications. In QEPAS, acoustic waves are generated between the prongs of a quartz tuning fork (QTF) by the absorption of modulated light from the gas molecules via non-radiative relaxation processes. QTFs are employed as piezoelectric-sensitive elements to transduce pressure waves in an electric signal. These quartz resonators are characterized by a good immunity to environmental acoustic noise due to their high quality factors (Q) and compact dimensions. Due to the sharp resonance, external noise sources outside of the resonator’s small bandwidth (~4 Hz at atmospheric pressure) do not influence the QTF signal. Suitable front-end electronics must be designed to read out the signal generated by the QTF and to optimize the signal-to-noise ratio (SNR), as well as the minimum detection limit of the gas concentration. A theoretical study of the SNR trend in a voltage-mode read-out of QTFs was presented [11], mainly focusing on the effects of (i) the noise contributions of both the QTF-equivalent resistor and the input bias resistor RL of the preamplifier; (ii) the operating frequency; and (iii) the bandwidth (BW) of the lock-in amplifier low-pass filter. As a result of the study, general guidelines for the choice of resistor RL and the most suitable operating frequency for the QEPAS system implementing the voltage-mode read-out of QTFs was derived. Furthermore, the proposed analysis allows for the study of the noise contributions at different bandwidths to optimize the acquisition time of QEPAS measurements.

Finally, we would like to thank all the authors for their submissions to this Special Issue; we very much appreciate their contributions. We also thank all the reviewers for dedicating their time and helping to ensure the quality of the submitted papers. Last but not least, we are grateful to the staff at the editorial office of *Micromachines*, and in particular, to Mr. Dikies Zhang, for their efficient assistance.

## Figures and Tables

**Figure 1:**
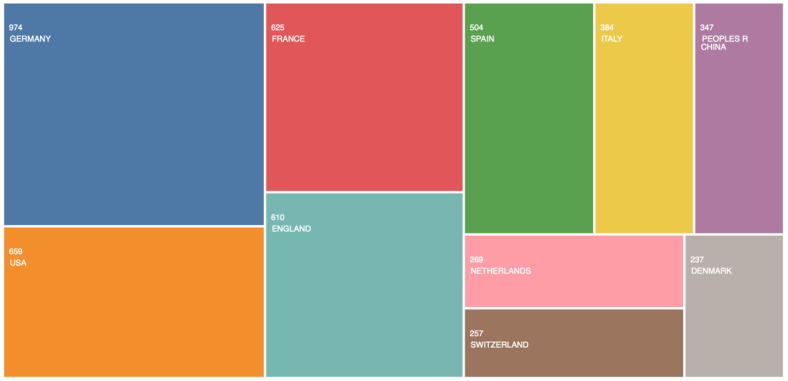
Chart presenting some of the countries of authors of publications relevant to the “nanophotonics” topic, according to a search in Clarivate Web of Science™; the search was refined by including only the 28 countries of European Community, plus Switzerland and the UK, in the “Countries/Regions” field. Here, only the countries with greater numbers of publications are shown. Since co-authors of a same publication may be affiliated with institutions in different countries (even outside Europe), the total of the numbers in the chart is greater than the number obtained from the refined search. Note: The areas on the chart are not strictly proportional to the values of each entry.
